# A Clinical–Radiomics Model for Predicting Axillary Pathologic Complete Response in Breast Cancer With Axillary Lymph Node Metastases

**DOI:** 10.3389/fonc.2021.786346

**Published:** 2021-12-21

**Authors:** Liangyu Gan, Mingming Ma, Yinhua Liu, Qian Liu, Ling Xin, Yuanjia Cheng, Ling Xu, Naishan Qin, Yuan Jiang, Xiaodong Zhang, Xiaoying Wang, Jingming Ye

**Affiliations:** ^1^ Breast Disease Center, Peking University First Hospital, Beijing, China; ^2^ Department of Radiology, Peking University First Hospital, Beijing, China

**Keywords:** breast cancer, radiomics, DCE-MRI, axillary lymph node, neoadjuvant chemotherapy

## Abstract

**Purpose:**

To develop a clinical–radiomics model based on radiomics features extracted from MRI and clinicopathologic factors for predicting the axillary pathologic complete response (apCR) in breast cancer (BC) patients with axillary lymph node (ALN) metastases.

**Materials and Methods:**

The MR images and clinicopathologic data of 248 eligible invasive BC patients at the Peking University First Hospital from January 2013 to December 2020 were included in this study. All patients received neoadjuvant chemotherapy (NAC), and the presence of ALN metastases was confirmed through cytology pre-NAC. The data from January 2013 to December 2018 were randomly divided into the training and validation sets in a ratio of 7:3, and the data from January 2019 to December 2020 served as the independent testing set. The following three types of prediction models were investigated in this study. 1) A clinical model: the model was built by independently predicting clinicopathologic factors through logistic regression. 2) Radiomics models: we used an automatic segmentation model based on deep learning to segment the axillary areas, visible ALNs, and breast tumors on post-NAC dynamic contrast-enhanced MRI. Radiomics features were then extracted from the region of interest (ROI). Radiomics models were built based on different ROIs or their combination. 3) A clinical–radiomics model: it was built by integrating radiomics signature and independent predictive clinical factors by logistic regression. All models were assessed using a receiver operating characteristic curve analysis and by calculating the area under the curve (AUC).

**Results:**

The clinical model yielded AUC values of 0.759, 0.787, and 0.771 in the training, validation, and testing sets, respectively. The radiomics model based on the combination of MRI features of breast tumors and visible ALNs yielded the best AUC values of 0.894, 0.811, and 0.806 in the training, validation, and testing sets, respectively. The clinical–radiomics model yielded AUC values of 0.924, 0.851, and 0.878 in the training, validation, and testing sets, respectively, for predicting apCR.

**Conclusion:**

We developed a clinical–radiomics model by integrating radiomics signature and clinical factors to predict apCR in BC patients with ALN metastases post-NAC. It may help the clinicians to screen out apCR patients to avoid lymph node dissection.

## 1 Introduction

Neoadjuvant chemotherapy (NAC) in breast cancer (BC) has the ability to downstage axillary lymph nodes (ALNs). Approximately 35%–63% of BC patients with positive ALNs can achieve an axillary pathologic complete response (apCR) post-NAC (1). Some studies have tried to perform sentinel lymph node (SLN) biopsy (SLNB) to avoid ALN dissection (ALND), but the detection rate of SLNs is low, and the false-negative rate (FNR) is relatively high. The Z1071 study (2) and the SENTINA study (3) showed that only after using a dual dye technique with both vital blue dye and radiolabeled colloid, and detecting at least 3 SLNs, can SLNB be considered safe in ALN-positive BC patients post-NAC. Some other studies (4–6) have shown that removal of the positive ALNs marked by clips pre-NAC while performing SLNB can significantly reduce the FNR of SLNB post-NAC. However, radiolabeled colloid and clip markers are not available in most hospitals in China, and it is not easy to achieve the requirement of detecting at least 3 SLNs; hence, there are many restrictions on SLNB among ALN-positive BC patients post-NAC. Currently in China, due to concerns about the high FNR of SLNB post-NAC, the recommended treatment for initial ALN-positive BC is ALND, which causes loss of opportunity to preserve the axilla in patients with apCR and increases the chance of suffering from ALND-related complications, such as limited shoulder mobility, wound infection, upper arm lymphedema, and paresthesia and pain in the surgical area (7, 8).

Routine imaging examinations, such as ultrasound (US), MRI, and positron emission tomography/CT (PET/CT), do not perform well in identifying the status of ALNs post-NAC (9–11). Recent studies have shown that MRI-based radiomics models can predict whether there will be ALN metastasis in BC patients (12–19). Some of these studies used the MRI radiomics features alone to predict the ALN status and achieved good performance (12, 13). Some other studies had combined the MRI radiomics features with other variables such as clinicopathologic characteristics (14, 16), pharmacokinetic parameters (15), kinetic curve pattern (17), and peritumoral MRI features (19) to build a combined model, which all showed better performance than the radiomics model alone. However, all the above studies used the MRI radiomics features of breast tumors to predict the status of ALN, and there were only a few studies that included the dedicated ALN MRI radiomics features in research (18, 20, 21). Yu et al. found that MRI radiomics features of ALN region were helpful for ALN metastasis prediction (18, 21). However, Samiei et al. found that dedicated ALN MRI radiomics features did not accurately predict ALN metastases in BC patients preoperatively (20). But the majority of patients enrolled in these three studies did not receive NAC, and none of the studies were specializing in the identification of the ALN status post-NAC. Therefore, the performance of MRI radiomics model in the identification of ALN status post-NAC in initial ALN-positive BC patients is still unclear. As mentioned above, the recommended post-NAC treatment for the axilla in these patients is ALND. Accurate identification of such patients with apCR can exempt ALND and avoid its related complications. Consequently, the purpose of this study was to add the dynamic contrast-enhanced MRI (DCE-MRI) features of ALNs into the radiomics analysis and to build a model that combines radiomics and clinicopathologic features to predict apCR.

## 2 Patients and Methods

This study was approved by the Ethics Committee of Peking University First Hospital [IRB number: 2019(170)]. The requirement of obtaining informed consent was waived, as it was a retrospective study.

### 2.1 Patient Inclusion

We identified female primary BC patients aged at least 18 years who were treated with NAC from January 2013 to December 2020 at our breast disease center. The inclusion criteria were as follows: i) patients who had confirmed primary BC by core needle biopsy (CNB); ii) CNB or fine-needle aspiration biopsy (FNAB)-confirmed metastases in ipsilateral ALN; iii) patients with disease staged T_1–4_N_1–2_M_0_; iv) DCE-MRIs were conducted pre- and post-NAC; v) patients who had completed at least 4 cycles of NAC; vi) ALND was conducted post-NAC; and vii) clinicopathologic data were available. The exclusion criteria were as follows: i) occult BC; ii) artifact on DCE-MRI; iii) patients had previously undergone axillary surgery; iv) patients with multifocal tumors; and v) patients with heterogeneous tumors.

#### 2.1.1 Clinicopathologic Data

All patients were staged according to the 8th edition of the American Joint Committee on Cancer Staging Manual (22). The estrogen receptor (ER) status, progesterone receptor (PR) status, and Ki67 expression were evaluated by immunohistochemistry (IHC) (23). ER/PR positivity (+) was defined as ≥1% of tumor cells with nuclear staining. Hormone receptor (HR) positivity (+) was defined as ER (+) and/or PR (+). Human epidermal growth factor receptor 2 (HER2) positivity (+) was determined according to the American Society of Clinical Oncology (ASCO) guidelines (24). Ki67 expression was defined as low (≤30%) or high (>30%) (25). All patients were divided into the following four subtypes according to HR and HER2 status: HR+HER2−, HR+HER2+, HR−HER2+, and HR−HER2− [triple negative (TN)]. Breast pathologic complete response (bpCR) was defined as the absence of residual invasive or *in situ* carcinoma in the surgical specimen (26). An apCR was defined as the complete absence of micrometastases and macrometastases in ALNs (26). The pathologic information of all patients was evaluated by the breast pathology team of our hospital and recorded in the patients’ medical records. Since these assessments were objective and followed accepted standards (23, 24), we did not reassess the pathology.

All patients underwent MRI and breast US pre- and post-NAC. CNB or FNAB was performed in suspicious ALNs. The NAC regimens were based on the National Comprehensive Cancer Network (NCCN) guidelines (27) or the guidelines of the Chinese Society of Clinical Oncology (CSCO) (25). All patients received anthracycline and/or taxane-based NAC regimens. For HER-2-negative patients, the regimens include TA (docetaxel/doxorubicin), TX (docetaxel/capecitabine), TAC (docetaxel/doxorubicin/cyclophosphamide), TC (docetaxel/cyclophosphamide), and TP (docetaxel/carboplatin) (25, 27). For HER2-positive patients, all the regimens were combined with anti-HER2 therapy including TCH (docetaxel/carboplatin/trastuzumab), TH (docetaxel/trastuzumab), and AC-TH (doxorubicin/cyclophosphamide–docetaxel/trastuzumab). All patients underwent breast-conserving surgery or total mastectomy and ALND after NAC (26, 27). The excised tissues were subjected to pathologic examination. The clinical tumor response to NAC was evaluated by the Response Evaluation Criteria in Solid Tumors version 1.1 (RECIST 1.1) (28) by MRI. With RECIST 1.1, we utilized the following classifications for therapeutic response: complete response (CR), primary tumor disappearance; partial response (PR), 30% or greater decrease in the longest diameter of the primary tumor; progressive disease (PD), 20% or greater increase in the longest diameter of the primary tumor; and stable disease (SD), tumors that did not show either sufficient shrinkage to be classified as PR or sufficient increase to be classified as PD (28).

The clinicopathologic data included age, menstrual status, HR status, HER2 status, initial clinical T stage (cT), initial clinical N stage (cN), histologic type, histologic grade, Ki67 expression, and clinical tumor response to NAC (cTR). Clinicopathologic data were obtained from patients’ medical records (Dr LG and LX, response for the data collection).

#### 2.1.2 MRI Acquisition Protocol

All MR images were acquired through a 3.0-T system machine with 8-channel breast coils (Signa Excite, GE Medical Systems, USA). After an intravenous injection of 0.1 mmol/kg of Gd-DTPA (Magnevist, Bayer Schering Pharma, Germany), a 20-ml saline solution was used to rinse at a flow rate of about 2 ml/s. The T1-weighted images (T1WI) of DCE-MRI included 1 pre-contrast and 8 post-contrast images with fat saturation. The third post-contrast T1WI of DCE-MRI post-NAC was analyzed for this study. The DCE-MRI parameters were as follows: repetition time (TR)/echo time (TE) = 4.53 ms/1.66 ms; field of view (FOV) = 34 cm × 34 cm; matrix = 384 × 384; slice thickness = 2.4 mm; intersection gap = 0 mm; bandwidth = 62.5 Hz; single scan time = 58 s; and single-phase scanning slices = 106.

#### 2.1.3 Dataset Allocation

**Figure 1:**
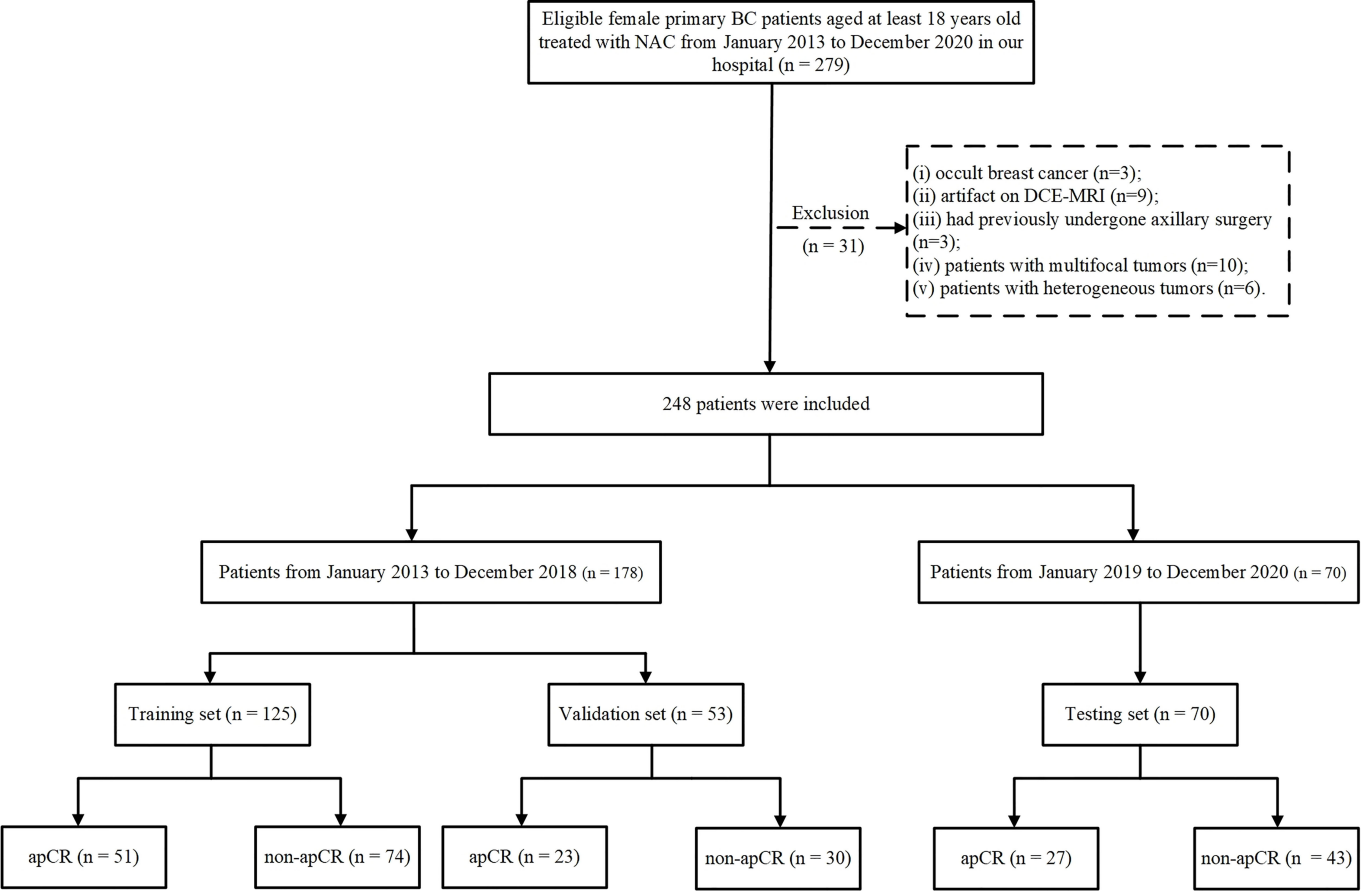
Flowchart of patient enrollment. BC, breast cancer; NAC, neoadjuvant chemotherapy; CNB, core needle biopsy; FNAB, fine-needle aspiration biopsy; ALN, axillary lymph node; ALND, axillary lymph node dissection; apCR, axillary pathologic complete response.

A total of 248 patients were included. The data from January 2013 to December 2018 were randomly divided into the training and validation sets in a ratio of 7:3. The data from January 2019 to December 2020 served as the independent testing set. The training set included 125 patients (51 apCR and 74 non-apCR cases), the validation set included 53 patients (23 apCR and 30 non-apCR cases), and the independent testing set included 70 patients (27 apCR and 43 non-apCR cases). The flowchart of patient enrollment is shown in **Figure 1**.

### 2.2 Development of a Clinical Prediction Model

The patients in the training set were divided into the apCR and non-apCR groups. Univariate and multivariable analyses were used to assess the difference in the clinicopathologic factors between the two groups, and then, the independent clinicopathologic predictive factors were used to develop the clinical model for the prediction of apCR through logistic regression.

### 2.3 Development of Radiomics Models

**Figure 2:**
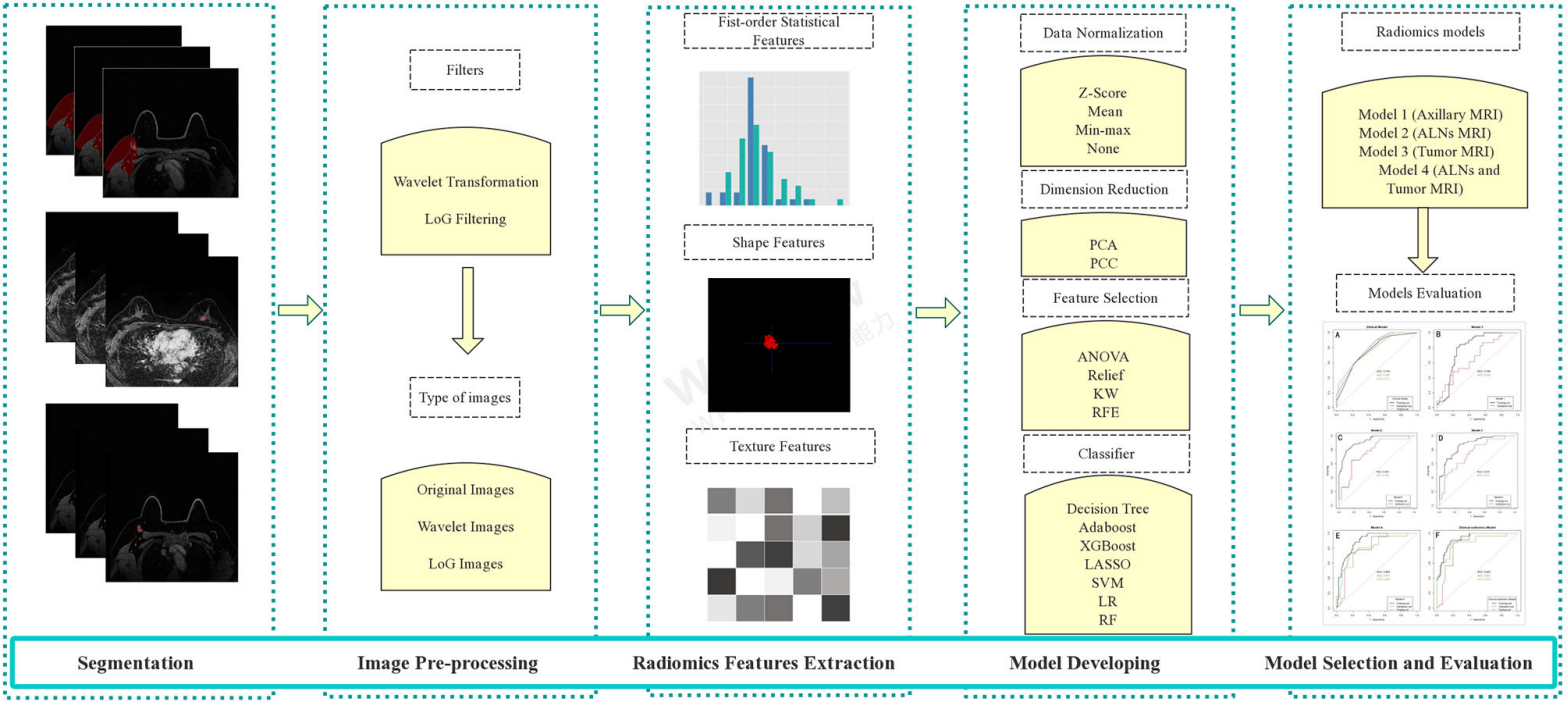
The workflow of MRI radiomics model development. LoG, Laplacian of Gaussian; PCA, principal component analysis; PCC, Pearson correlation coefficient; KW, Kruskal–Wallis; RFE, recursive feature elimination; SVM, support vector machine; LR, logistic regression; LASSO, least absolute shrinkage and selection operator; RF, random forest; ALNs, axillary lymph nodes.

The radiomics analysis process in this study was as follows: i) segmentation of the regions of interest (ROIs) on MRI; ii) image pre-processing; iii) radiomics feature extraction; and iv) radiomics model building and evaluation. The workflow of MRI radiomics models developing is shown in **Figure 2**.

#### 2.3.1 Segmentation of the Regions of Interest on MRI

**Figure 3:**
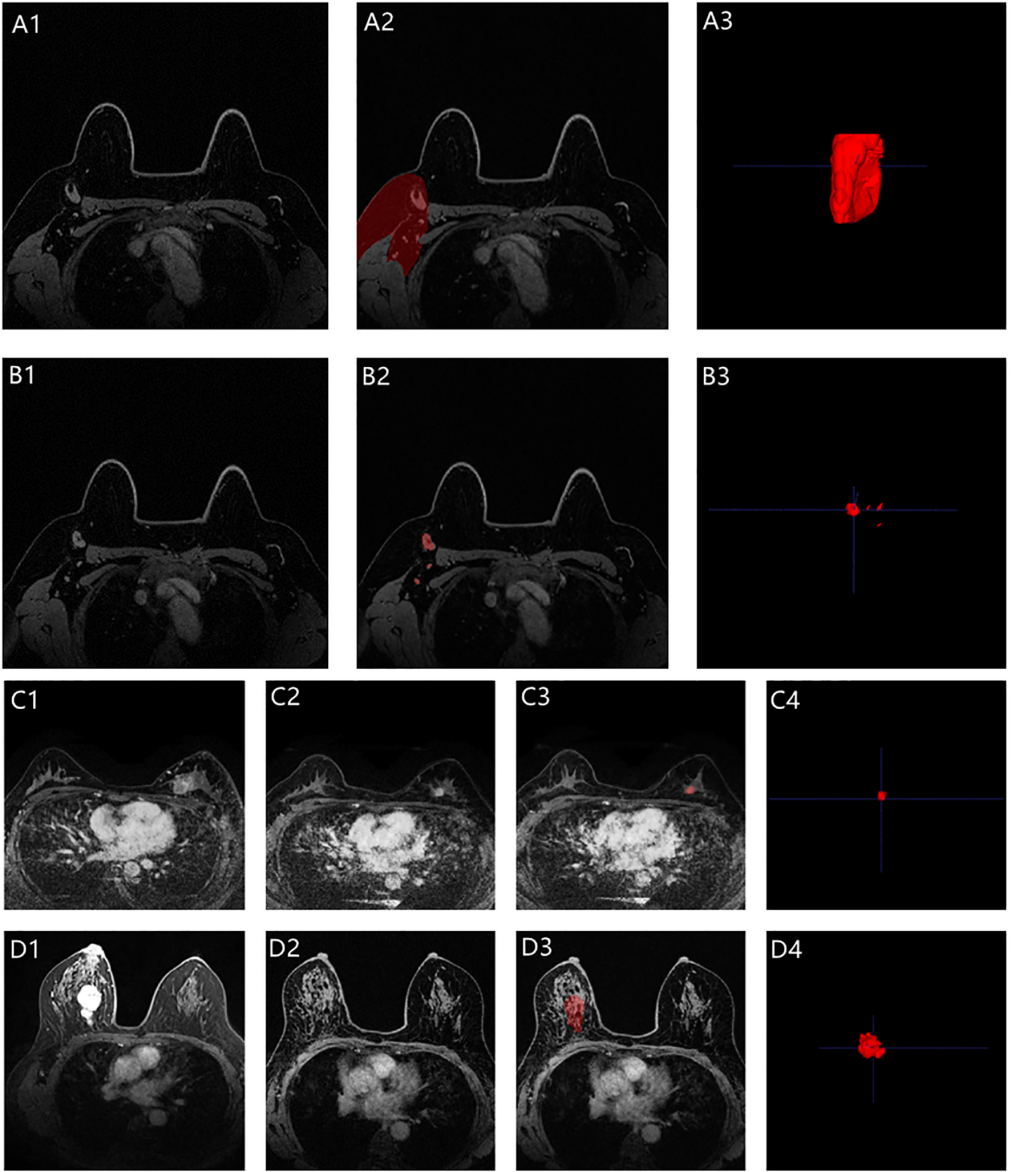
Representative images segmentation (A1–A3 for the axilla; B1–B3 for the visible ALNs; C1–C4 for the primary tumor with non-bpCR; D1–D4 for the primary tumor with bpCR; C1 and D1 were the pre-NAC MRIs, while C2 and D2 were the post-NAC MRIs). A3, B3, and C4 were automatically segmented by the deep learning model. The breast tumor bed (D3) was manually segmented based on the anatomical landmarks according to the pre-NAC MRI (D1).

To extract the imaging features of ALNs, we designed two types of ROIs. One was the axillary area on MRI, and the other was all visible ALNs on MRI. We also included features of the primary tumor in the analysis. A previously trained 3-dimensional (3D) U-Net segmentation model based on deep learning in Python (v 3.6.0, https://www.python.org/) was used to automatically segment the breast tumor, axillary area, and visible ALNs on post-NAC DCE-MRI (29). While segmenting the axillary area, the upper boundary was the top of the scan range; the lower boundary was approximately flat with the fourth rib; the posterior boundary was roughly composed of the anterior edge of the latissimus dorsi, teres major, and subscapularis; and the medial border was mainly the serratus anterior muscle (including the lymphatic adipose tissue between the pectoralis major and minor muscles and behind the pectoralis minor muscle). If there was no visible tumor on the DCE-MRI post-NAC, the radiologists segmented the tumor bed manually by the presence of tumor bed fibrosis and/or anatomical landmarks according to the pre-NAC MRI (radiologist NQ and radiologist XW with 15 and 17 years of experience in radiological diagnosis, respectively, response for the manually segmentation). All the radiologists were blinded to the patients’ pathologic outcomes. They separately performed manual segmentation for all cases that needed manual segmentation. The Dice score between radiologists NQ and XW was calculated as follows: $Dice=\frac{2(X\cap Y)}{X+Y}$ , where *X* and *Y* denote the number of pixels in the segmented images of Radiologists A and B, respectively, and *X* ∩ *Y* denotes the number of pixels in the overlapping part of the two radiologists’ segmented images. In this study, in all patients who had not achieved an apCR, at least one lymph node could be seen in the axillae. However, among the patients who achieved apCR, there were very few patients with no visible lymph nodes in the axillae. At this time, we would determine the approximate location of the ALN in the post-NAC MRI based on the position of the ALNs shown in previous MRIs according to anatomical landmarks and then use the deep learning model to generate a substitute ROI with an average volume of all the segmented lymph nodes in the corresponding area. All the automatically segmented images were checked by two dedicated breast radiologists on ITK-SNAP Version 3.6.0 (www.itksnap.org) and manually modified if necessary (radiologists MM and XW, response for the modified). A total of 30 random patients were selected, and radiologists MM and XW checked the images of these 30 patients separately and modified them if necessary. The Dice score between radiologists MM and XW in these 30 patients was also calculated after checking the images. Examples of segmentation are shown in **Figure 3**. If there were disagreements between the radiologists during the image segmentation process, they reach a consensus according to the viewpoint of the majority of radiologists who participated in this study.

#### 2.3.2 Image Pre-Processing and Radiomics Feature Extraction

Three types of images were used for analysis in this study, namely, “Original Images,” “Laplacian of Gaussian (LoG) Images,” and “Wavelet Images.” “Original Images” were the images without any transformation, while “LoG Images” were the images with LoG filtered, and “Wavelet Images” were the images with wavelet transformation. LoG filter was mainly used to enhance image edge detection, while wavelet transformation was used for image denoising and improving image quality. The LoG filter was applied to the original images to obtain fine to coarse textures (filter width: fine, σ = 1.0; medium, σ = 3.0; coarse, σ = 5.0). So there were 3 types of LoG images. The original images underwent a three-dimensional (i.e., x, y, and z directions) wavelet transformation through the *PyWavelet* package on Python. Each image was filtered by a high band-pass filter or low band-pass in the three directions, thus resulting in 8 combinations of different decompositions: LLH, LHL, HLL, LHH, HHL, HLH, HHH, and LLL (H means high, and L means low). Then the PyRadiomics software package in Python (https://github.com/radiomics/pyradiomics) was used to extract radiomics features. All characteristics were recorded and stored in a quantitative form. A total of 14 types of shape features, 18 types of first-order statistical features, and 70 types of texture features [n = 24 Gray Level Co-occurrence Matrix (GLCM) + 16 Gray Level Run Length Matrix (GLRLM) + 16 Gray Level Size Zone Matrix (GLSZM) + 14 Gray Level Dependence Matrix (GLDM)] were used for analysis in our study (**Supplementary Table 1**). The shape features were only extracted from the “Original Images,” while the first-order statistical and texture features were extracted from all three types of images. Thus, the extracted features contained 14 shape features, 216 first-order statistical features [(1 Original Images + 3 LoG Images + 8 Wavelet Images) × 18], and 840 texture features [(1 Original Images + 3 LoG Images + 8 Wavelet Images) × 70]. All shape features, first-order statistical features, and texture features were extracted from the visible ALNs and primary tumors. Among the visible ALNs, the shape features were extracted from the lymph node most suspected of showing metastasis, and when none of the lymph nodes were suspected of showing metastasis, the largest lymph node among them was designated as the lymph node for extracting shape features. While extracting first-order statistical features and texture features, the visible ALNs were extracted as a whole. While extracting radiomics features in the axillary area, we only extracted the first-order features and texture features because we believed that the shape features of the axillary area did not represent the characteristics of ALNs.

#### 2.3.3 Radiomics Model Development and Evaluation

In the training set, the radiomics models were developed by following the four processes: data normalization (four methods), dimension reduction (two methods), feature selection (four methods), and classification (seven methods) (**Tables 1**, **S1** in the **Supplementary Material**). All possible combinations of the methods were used for building radiomics models. Several methods in each process were used in order to provide more choices for the model building and to choose more suitable modeling methods. Four types of radiomics models were built, namely, a model based on the MRI features of the axillary region (Model 1), a model based on the MRI features of visible ALNs (Model 2), a model based on the MRI features of breast tumor (Model 3), and a model based on the combination of the breast tumor and the axillary region or visible ALN MRI features, depending on Model 1 and Model 2, whichever would perform better (Model 4). The performance of all the radiomics models for predicting apCR was investigated in the training and validation sets. The radiomics model of each type with the best performance between the training and validation sets would be selected as the final model of each type. When several models had the same or quite similar prediction performance, a model that included the fewest features was chosen in order to reduce the complexity of the model and the risk of non-generalization. All the works related to radiomics model development and evaluation were completed through Feature Explorer Pro (FAEPro, V 0.3.4) in Python (v 3.6.0) (30).

**Table 1:** Alternative methods at every step of the modeling process.

Modeling Steps	Methods
Normalization	None
	Mean
	Z-score
	Min–max
Dimension reduction	Pearson correlation coefficient (PCC)
	Principal component analysis (PCA)
Feature selection	Recursive feature elimination (RFE)
	ANOVA
	Kruskal–Wallis test (KWT)
	Relief
Classification	Least absolute shrinkage and selection operator (LASSO)
	Random forest (RF)
	Support vector machine (SVM)
	Decision tree
	XGBoost
	Adaboost
	Logistic regression (LR)

### 2.4 Development of a Clinical–Radiomics Model

A clinical–radiomics model was built by integrating MRI features used in the best radiomics model and clinical factors used in the clinical model. The clinical–radiomics model was developed in the training set and then evaluated in the validation and testing sets.

### 2.5 Statistical Analysis

The chi-square test was used to compare the categorical variables between the training and validation sets. The variables between the apCR and non-apCR groups in the training set were also compared by chi-square test and the variables with significant differences (p < 0.05) were submitted to the multivariate analysis using logistic regression, and the hazard ratios (HRs) and 95% CIs were calculated. After multivariate analyses, the clinical model was built with the independent predictive clinical factors (*p* < 0.05) in the training set by logistic regression. The predictive performance of all models was assessed using a receiver operating characteristic (ROC) curve analysis and by calculating the area under the curve (AUC). The sensitivity, specificity, accuracy, positive predictive value (PPV), and negative predictive value (NPV) were calculated at a cutoff value that maximized the value of Youden’s index in the training set. Calibration curves were used to show the agreement between the predicted probability of apCR and real observed probability in the clinical–radiomics model. Decision curve analysis (DCA) was used to evaluate the clinical decision value of each model. For all statistics, *p* < 0.05 was considered statistically significant, and all tests were two-tailed. R software (version 4.1.0, www.r-project.org) was used to conduct all statistical analyses.

## 3 Results

### 3.1 Characteristics of the Patients

The clinical characteristics of the entire training, validation, and testing sets are shown in **Table 2**. All characteristics were not significantly different between the training and validation sets (*p* > 0.05). An apCR was observed in 101 (40.7%) cases (training set, n = 51; validation set, n = 23; and testing set, n = 27).

**Table 2:** Clinicopathologic characteristics of patients.

Characteristic	No. (%)				*p*-Value
	Entire set (n = 248)	Training set (n = 125)	Validation set (n = 53)	Testing set (n = 70)	
Age, years					0.44
≤40	49 (19.8)	25 (20.0)	8 (15.1)	16 (22.9)	
>40	199 (80.2)	100 (80.0)	45 (84.9)	54 (77.1)	
Menopausal					0.51
Premenopausal	134 (54.0)	68 (54.4)	26 (49.1)	40 (57.1)	
Postmenopausal	114 (46.0)	57 (45.6)	27 (50.9)	30 (42.9)	
Histological type					0.68
Invasive ductal carcinoma	232 (93.5)	114 (91.2)	50 (94.3)	68 (97.1)	
Invasive lobular carcinoma or others	16 (6.5)	11 (8.8)	3 (5.7)	2 (2.9)	
Clinical T stage					0.61
1	26 (10.5)	16 (12.8)	6 (11.3)	4 (5.7)	
2	172 (69.4)	80 (64.0)	39 (73.6)	53 (75.7)	
3	39 (15.7)	20 (16.0)	6 (11.3)	13 (18.6)	
4	11 (4.4)	9 (7.2)	2 (3.8)	0 (0)	
Clinical N stage					0.76
1	170 (68.5)	82 (65.6)	36 (67.9)	52 (74.3)	
2	78 (31.5)	43 (34.4)	17 (32.1)	18 (25.7)	
HR					0.60
Negative	107 (43.1)	56 (44.8)	26 (49.1)	25 (35.7)	0.27
Positive	141 (56.9)	69 (55.2)	27 (50.9)	45 (64.3)	
HER2					
Negative	134 (54.0)	67 (53.6)	31 (58.5)	36 (51.4)	
Positive	114 (46.0)	58 (46.4)	22 (41.5)	34 (48.6)	
Subtypes					0.30
HR+HER2−	89 (35.9)	48 (38.4)	17 (32.1)	24 (34.3)	
HR+HER2+	52 (21.0)	21 (16.8)	10 (18.9)	21 (30.0)	
HR−HER2+	62 (25.0)	37 (29.6)	12 (22.6)	13 (18.6)	
HR−HER2−	45 (18.1)	19 (15.2)	14 (26.4)	12 (17.1)	
Histological grade					0.26
1, low	8 (3.2)	6 (4.8)	1 (1.9)	1 (1.4)	
2, intermediate	132 (53.2)	75 (60.0)	27 (50.9)	30 (42.9)	
3, high	108 (43.6)	44 (35.2)	25 (47.2)	39 (55.7)	
Ki67					0.75
≤30%	71 (28.6)	36 (28.8)	14 (26.4)	21 (30.0)	
>30%	177 (71.4)	89 (71.2)	39 (73.6)	49 (70.0)	
Clinical tumor response					0.50
SD	31 (12.5)	17 (13.6)	4 (7.5)	10 (14.3)	
PR	192 (77.4)	96 (76.8)	45 (84.9)	51 (72.9)	
CR	25 (10.1)	12 (9.6)	4 (7.6)	9 (12.8)	
Axillary pathologic complete response					0.75
Yes	101 (40.7)	51 (40.8)	23 (43.4)	27 (38.6)	
No	147 (59.3)	74 (59.2)	30 (56.6)	43 (61.4)	

HR, hormone receptor; HER2, human epidermal growth factor receptor 2; SD, stable disease; PR, stable disease; CR, complete response; T, tumor; N, node; p, χ^2^ test between the training and validation cohorts.

### 3.2 The Clinical Model for Predicting an Axillary Pathologic Complete Response

**Figure 4:**
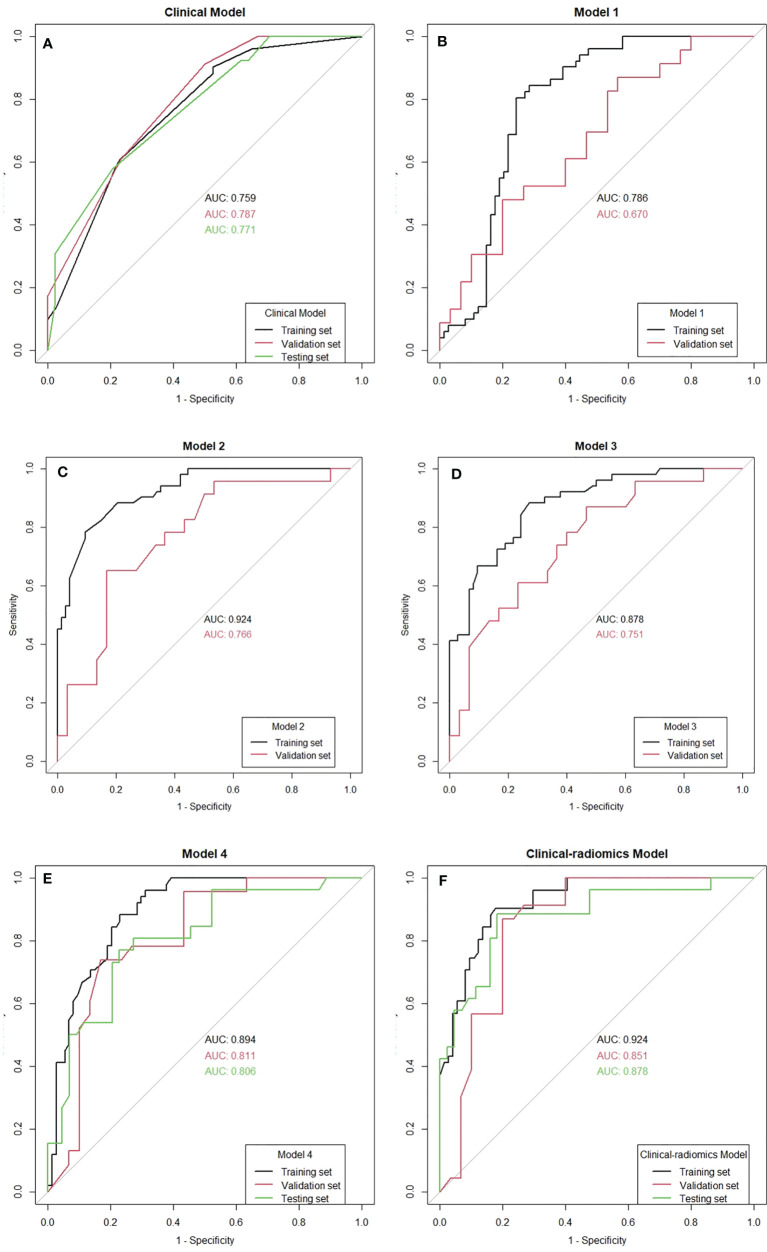
The receiver operating characteristic (ROC) curves of all the models. AUC, area under the curve.

On univariable analysis, cN, HR status, and cTR were significantly associated with apCR (*p* < 0.05, **Table 3**) in the training set. After multivariate analyses based on logistic regression, cN, HR status, and cTR were independent predictors and were selected to build the clinical model to predict apCR (**Table 3**). The clinical model yielded AUC values of 0.759 in the training set, 0.787 in the validation set, and 0.771 in the testing set for predicting an apCR (**Figure 4A**).

**Table 3:** Univariable and multivariate analyses of apCR post-NAC in relation to clinicopathologic characteristics in the training cohort.

Characteristic	Axillary pathologic complete response No. (%)		*p* ^1^ value	OR	95% CI95% CI	*p* ^2^ value
	No (n = 74)	Yes (n = 51)				
Age, years						
≤40	16 (21.6)	9 (17.6)	0.59			
>40	58 (78.4)	42 (82.4)				
Menopausal						
Premenopausal	39 (52.7)	29 (56.9)				
Postmenopausal	35 (47.3)	22 (43.1)	0.65			
Histological type						
Invasive ductal carcinoma	67 (90.5)	47 (92.2)				
Invasive lobular carcinoma or others	7 (9.5)	4 (7.8)	1			
Clinical T stage						
1	9 (12.2)	7 (13.7)				
2	47 (63.5)	33 (64.7)				
3	12 (16.2)	8 (15.7)				
4	6 (8.1)	3 (5.9)	0.96			
Clinical N stage						
1	38 (51.4)	44 (86.3)	<0.001	5.58	2.21–15.98	<0.001
2	36 (48.6)	7 (13.7)				
HR						
Negative	24 (32.4)	32 (62.7)	<0.001	3.03	1.36–6.93	0.007
Positive	50 (67.6)	19 (37.3)				
HER2						
Negative	43 (58.1)	24 (47.1)				
Positive	31 (41.9)	27 (52.9)	0.22			
Histological grade						
1, low	5 (6.7)	1 (2.0)				
2, intermediate	46 (62.2)	29 (56.9)				
3, high	23 (31.1)	21 (41.1)	0.29			
Ki67						
≤30%	26 (35.1)	10 (19.6)				
>30%	48 (64.9)	41 (80.4)	0.06			
Clinical tumor response						
SD/PR	71 (95.9)	42 (82.4)				
CR	3 (4.1)	9 (17.6)	0.03	5.00	1.25–26.17	0.033

HR, hormone receptor; HER2, human epidermal growth factor receptor 2; SD, stable disease; PR, stable disease; CR, complete response; T, tumor; N, node; apCR, axillary pathologic complete response; p^1^ value, χ^2^ test between the apCR and non-apCR cohorts; p^2^ value, multivariate analysis result.

### 3.3 Radiomics Models for Predicting an Axillary Pathologic Complete Response

The mean Dice score between radiologists NQ and XW in manually segmented cases was 0.864, and the mean Dice score between radiologists MM and XW in the 30 automatically segmented cases after the manual correction was 0.948, indicating good image segmentation consistency between researchers. After dimension reduction of feature matrices, the feature selector selected the top 20 features of each model for modeling. The outputs of radiomics models were the predicted probabilities of patients belonging to the two categories (i.e., apCR or non-apCR). We called the predicted probabilities of apCR “radiomics signatures” and used them to perform the ROC analysis. Finally, Models 1, 2, and 3 using six, nine, and six features, respectively, yielded the best performance (i.e., AUC value) in predicting an apCR. Model 1, Model 2, and Model 3 yielded AUC values of 0.786, 0.924, and 0.878 in the training set and 0.670, 0.766, and 0.751 in the validation set, respectively (**Figures 4B–D**). The performances of Model 2 and Model 3 were similar, and both of them were better than Model 1. Hence, we built Model 4 based on the combination of MRI features of breast tumors and ALNs. Finally, Model 4 with six features (four from the breast tumor and two from the ALNs) yielded the best performance. The AUC values of Model 4 were 0.894 in the training set and 0.811 in the validation set. Therefore, Model 4 was the best-performing radiomics model and was selected as the final radiomics model. We tested Model 4 in the independent testing set, and it yielded an AUC value of 0.806 for predicting apCR (**Figure 4E**). The modeling processes of the 4 radiomics models are shown in **Table 4**, and the final selected radiomics features of the 4 radiomics models are listed in **Table 5**.

**Table 4:** Construction process of the 4 radiomics models.

Radiomics Processes	Model 1 (Axillary MRI)	Model 2 (ALN MRI)	Model 3 (Tumor MRI)	Model 4 (ALNs and Tumor MRI)
Data normalization	None	None	Min–max	Mean
Dimension reduction	PCA	PCC	PCC	PCC
Feature selection	KWT	RFE	ANOVA	KWT
Classification	Adaboost	Adaboost	Adaboost	RF

ALNs, axillary lymph nodes; PCA, principal component analysis; PCC, Pearson correlation coefficient; RFE, recursive feature elimination; KWT, Kruskal–Wallis test; RF, random forest.

**Table 5:** Key features used in the four radiomics models.

Model name	Key features
Model 1	PCA_feature_5
	PCA_feature_6
	PCA_feature_53
	PCA_feature_58
	PCA_feature_79
	PCA_feature_89
Model 2	wavelet-HHH_firstorder_Mean
	wavelet-HLH_firstorder_Mean
	wavelet-LHH_glszm_SmallAreaLowGrayLevelEmphasis
	wavelet-LHL_firstorder_Skewness
	wavelet-HHL_firstorder_Skewness
	wavelet-LLH_glcm_ClusterShade
	log-sigma-3-0-mm-3D_glcm_ClusterShade
	log-sigma-5-0-mm-3D_firstorder_Skewness
	log-sigma-5-0-mm-3D_gldm_LargeDependenceLowGrayLevelEmphasis
Model 3	original_shape_SurfaceVolumeRatio
	original_shape_Sphericity
	wavelet-LHH_glcm_Correlation
	wavelet-LHH_glcm_MCC
	log-sigma-3-0-mm-3D_firstorder_Skewness
	log-sigma-5-0-mm-3D_firstorder_Skewness
Model 4	mass-original_shape_SurfaceVolumeRatio
	mass-wavelet-LHL_firstorder_Median
	mass-wavelet-LLH_firstorder_RootMeanSquared
	Lymphnode-wavelet-HHH_firstorder_Mean
	mass-log-sigma-5-0-mm-3D_firstorder_90Percentile
	Lymphnode-wavelet-LHH_glszm_SmallAreaLowGrayLevelEmphasis

H, high; L, low; GLCM, Gray Level Co-occurrence Matrix; GLSZM, Gray Level Size Zone Matrix; GLDM, Gray Level Dependence Matrix.

### 3.4 The Clinical–Radiomics Model for Predicting an Axillary Pathologic Complete Response

**Figure 5:**
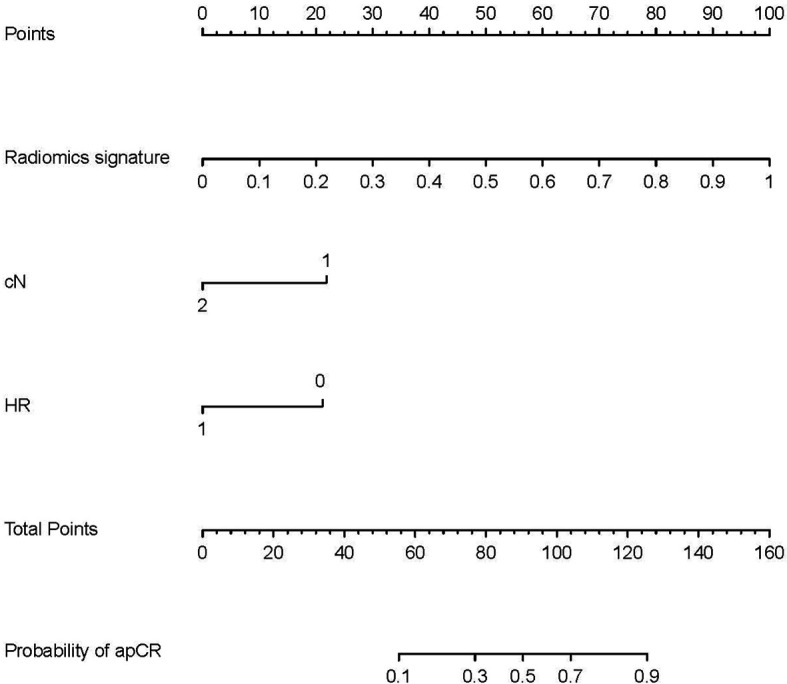
Nomogram for prediction of apCR. The different values of each variable correspond to a point at the top of the graph, and the sum of the points of all variables corresponds to total points. The line from the total point to the bottom is the probability of apCR. HR, hormone receptor: 0 means negative, 1 means positive. cN, clinical N stage; apCR, axillary pathologic complete response.

**Figure 6:**
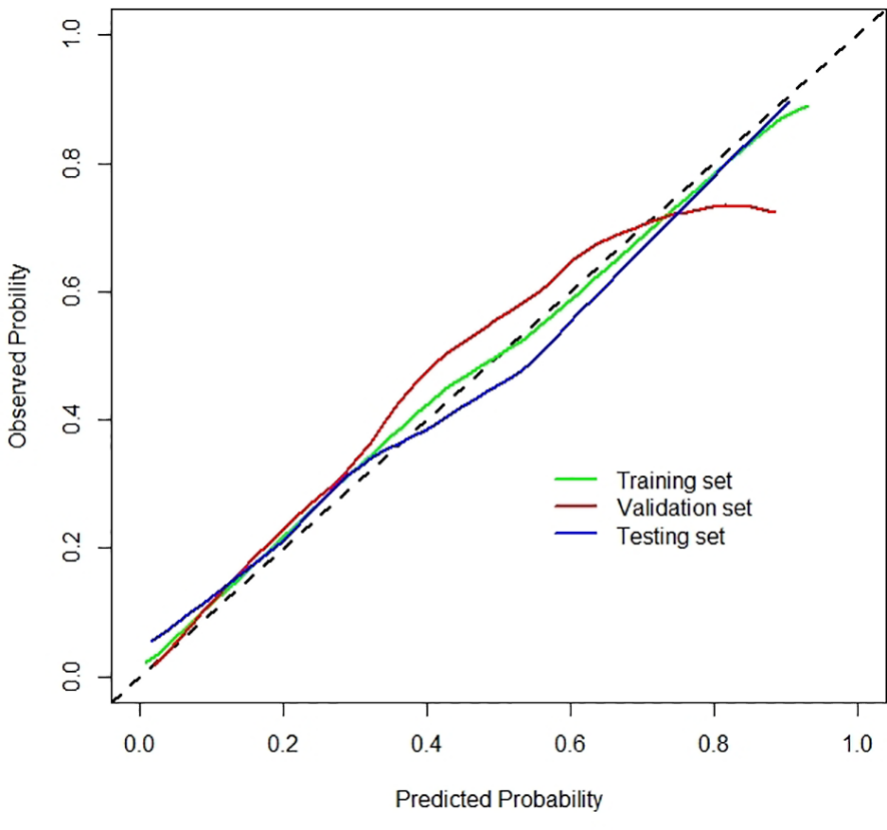
Calibration curves of the clinical–radiomics model for the training, validation, and testing sets.

Radiomics signatures provided by Model 4 showed significant differences (*p* < 0.001) between the apCR and non-apCR patients in the training set. After multivariate logistic regression with the independent clinical predictors (cN, HR status, and cTR), radiomics signatures, HR status, and cN were independent predictors of apCR (**Supplementary Table 2**). The clinical–radiomics model was built based on these independent predictors through logistic regression in the training set. The clinical–radiomics model yielded AUC values of 0.924, 0.851, and 0.878 in the training set, validation set, and testing set, respectively (**Figure 4F**). To show Model 4 more intuitively and to increase its clinical applicability, we established a nomogram to display Model 4 (**Figure 5**). The calibration curves showed good consistency between the predicted probability by the nomogram and the observed probabilities in the three sets (**Figure 6**).

### 3.5 Comparison of the Performance of Models

**Figure 7:**
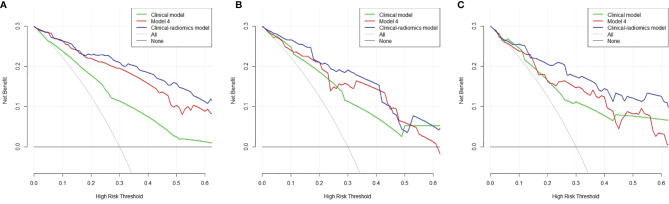
Decision curve analysis of the three models in the training **(A)**, validation **(B)**, and testing **(C)** sets. The x-axis indicates the threshold probability, while the y-axis indicates the net benefit. The gray line indicates the hypothesis that all the patients achieved an apCR, and the black line indicates the hypothesis that none of the patients achieved an apCR. apCR, axillary pathologic complete response.

The optimal cutoff values for predictive apCR of each model were determined in the training set, which maximized the value of Youden’s index. The cutoff values of the clinical model, Model 4, and the clinical–radiomics model were 0.486, 0.640, and 0.361, respectively. Based on the cutoff values, the accuracy, sensitivity, specificity, PPV, and NPV of the clinical model, Model 4, and clinical–radiomics model were calculated in the training, validation, and testing sets (**Table 6**). Taken together, the clinical–radiomics model performed the best in the three sets, and Model 4 was slightly better than the clinical model. The DCA showed that the net benefit of using the clinical–radiomics model to predict an apCR was greater than that of the two other models in the training, validation, and testing sets (**Figure 7**).

**Table 6:** The performance of the three models in the training, validation, and testing sets.

Models	AUC	Sensitivity	Specificity	Accuracy	PPV	NPV
Training set						
Clinical model	0.759	0.608	0.770	0.704	0.646	0.740
Model 4	0.894	0.882	0.770	0.816	0.726	0.905
Clinical–radiomics model	0.924	0.902	0.824	0.856	0.780	0.924
Validation set					
Clinical model	0.787	0.609	0.767	0.698	0.667	0.719
Model 4	0.811	0.739	0.833	0.792	0.773	0.806
Clinical–radiomics model	0.851	0.870	0.800	0.830	0.769	0.889
Testing set					
Clinical model	0.771	0.577	0.795	0.714	0.625	0.761
Model 4	0.806	0.731	0.773	0.757	0.655	0.829
Clinical–radiomics model	0.878	0.885	0.750	0.800	0.676	0.917

AUC, area under the curve; PPV, positive predictive value; NPV, negative predictive value.

## 4 Discussion

In this study, we designed three types of models to predict apCR in initial ALN-positive BC patients post-NAC. We found that the clinical–radiomics model that integrated clinicopathologic factors and MRI signature had better predictive performance than the clinical model or radiomics model alone. Previous studies (31–34) have shown that patients who had BCs with HER2 positivity, TN subtypes, high Ki67 expression, higher histological grade, lower initial cN, and good tumor response to NAC were more likely to achieve apCR. All these characteristics were included in our study, and we found that cN, HR status, and clinical tumor response to NAC were significantly associated with apCR on univariable analysis. This is consistent with previous research. After multivariate analyses, cN, HR status, and clinical tumor response to NAC were all independent predictors. The clinical model was built using these independent predictors, and the model performed moderately on the training, validation, and testing sets.

Unlike many previous studies that used MRI features of breast tumors to predict the status of ALN (12–17, 19), we included dedicated ALN MRI features for research in the present study. We designed two types of ROIs to include the radiomics features of ALNs: one was the axillary area on the MRI, and the other was the visible lymph nodes in the axillary region. In addition to ALNs, the axillary area included features of other tissues within the axilla; thus, it was not particularly precise but was easy to perform image segmentation. The visible ALNs more accurately contained the radiomics features of ALNs, but it was not easy to perform image segmentation. Since the response of ALNs to NAC is closely related to the response of primary breast tumors to NAC (31, 32), we also included MRI features of primary breast tumors in the analysis. We believed that the post-NAC MRI features of the tumor can reflect the tumor’s response to NAC, and we hypothesized that there are differences among those tumors with different responses to NAC.

Before extraction of the radiomics features, accurate segmentation of the ROI is a key step in radiomics research. Segmentation methods include manual, semi-automatic, and fully automatic methods (35). Manual segmentation is time-consuming, and it increases the labor burden. In this study, we used a deep learning model to automatically segment the ROIs, which was time-saving and labor-saving. To segment the ROIs more accurately, radiologists checked the automatically segmented images and made corrections whenever necessary; hence, the reliability of the research was assured.

We built four radiomics models through various combinations of the features extracted from each ROI. Finally, Model 1 yielded an AUC value of 0.673 in the validation set, its predictive performance was poor, and it was not better than the clinical model. The performances of Model 2 and Model 3 were similar to those of the clinical model in the validation set, and both of them were better than Model 1. These results suggested that the radiomics features of breast tumors or ALNs can be used to predict apCR. Therefore, we discarded Model 1 and built Model 4 based on the combination of MRI features of breast tumors and ALNs. Our purpose was to detect whether the combined model could improve the prediction performance. The result obtained indicated that Model 4 performed better than all the other radiomics models and the clinical model. Therefore, Model 4 was selected as the final radiomics model.

After integration of clinical factors and MRI radiomics signature to build a combined model, the clinical–radiomics model had a significantly improved predictive ability in all the three sets. The DCA also shows that the net benefit of using the clinical–radiomics model to predict an apCR was greater than that of using the radiomics model and clinical model in the three sets. All these results indicate that the clinical–radiomics model can help us to more accurately identify patients who have achieved an apCR.

Few previous studies had included dedicated ALN MRI radiomics features in the prediction of ALN metastasis in BC patients (18, 20, 21). Yu et al. found that radiomics features extracted from ALNs were better than radiomics features extracted from primary tumors for ALN status identification, and the clinical–radiomics nomogram accurately predicted ALN metastasis in the development and validation cohorts (AUC, 0.92 and 0.90, respectively) (18). Another study also conducted by Yu et al. showed that the ALN-tumor radiomics signature for ALN status prediction showed a high prediction quality with AUCs of 0.88, 0.87, and 0.87 in the training cohort, external validation cohort, and prospective-retrospective validation cohort, respectively. The model incorporating tumor and ALN MRI radiomics features and clinicopathologic characteristics further improved the performance for ALN status prediction (21). Our results were similar to those of the two studies, but the difference was that the patients included in their studies were treated with or without NAC. In addition, the primary endpoint of these two studies was not predicting an apCR, while all the patients included in our study had ALN metastasis confirmed by pathology pre-NAC. The current recommended treatment for these patients post-NAC is still ALND. For patients with ALN negative, SLNB can be used to determine the status of the axillary post-NAC. That is to say, it is more necessary to build a model to predict the status of ALN in initial ALN-positive patients post-NAC. Our research filled this gap. There is also a study that found that dedicated axillary MRI radiomics features did not accurately predict ALN metastases in BC patients preoperatively (20). However, the sample size of this study was too small, and the conclusion needs further verification.

The best radiomics model, Model 4, contained one shape feature, four first-order statistical features, and one texture feature. The shape features include two- or three-dimensional descriptors derived from ROI and are regarded as vital features to evaluate the characteristics of tumors (35, 36). The first-order statistical features indicate the statistical value of the image intensity and are used to evaluate the homogeneous patterns and variability in an image (35, 36). The texture features indicate the spatial inter-dependency or co-occurrence of information across neighboring voxels of the image and are used to evaluate the intertumoral heterogeneity (35, 36). The shape features, first-order statistical features, and texture features were widely used in the studies on the identification of ALN status (12–18, 35, 36). Previous studies had reported that the texture features could reflect the breast tumor molecular subtypes and lymphocyte infiltration (37). The texture features could reflect the BC heterogeneity combined with the first-order statistical features (38). And the genetic characteristics could be predicted by the shape and texture features (39). The alliance of these radiomics features could detect the BC microenvironment (21). In other words, MRI radiomics features can reflect the biological behavior of breast tumors. All this biological information of BC can be used to predict tumor metastasis and NAC response (40, 41). This may be the reason why we can use MRI radiomics features to predict an apCR. Due to the large number of MRI radiomics features, as well as the difference in the purposes and methods used in these studies, the specific radiomics features ultimately selected for modeling were mostly different. But the shape feature of surface volume ratio and the texture feature of GLSZM included in our study has already been used to predict ALN status previously (18, 21). Our research also proves that the first-order statistical features can be used to predict apCR, which is consistent with previous research.

Our study has some limitations. First, this was a retrospective study at a single center with a small sample size, and further verification is needed by multicenter studies in the future. Second, the biological behaviors of different molecular subtypes of BC are different, but we did not analyze different molecular subtypes separately. In the future, we will collect more cases and differentiate subtypes to perform subtype-specific research. Third, due to the special position of breast MRI examination, the axillary area in some patients may not be completely covered by breast MRI, which may affect the radiomics analysis. As an exploratory research, this study is a preliminary attempt. In the future, we will consider covering the axillary area as much as possible by changing the examination position or using special axillary coils. Fourth, we regarded the pathologic results of ALND as the gold standard for identifying apCR, and the visible ALNs on the MRI were assessed as a whole. Currently, we could not achieve a one-to-one correspondence between ALNs and pathologic results. In the future, we may be able to use measures, such as placing marker clips in the ALNs to achieve this purpose. Fifth, only the third phase of post-contrast DCE-MRI was included for radiomics analysis. Adding more MRI sequences may improve the predictive performance of radiomics models. We plan to include more MRI sequences for study in the future. Finally, only the pre-NAC MRI was included in the study. The pre-NAC MRI features were often used to predict whether the primary breast tumor would achieve a complete pathologic response in previous studies (42, 43). We believe that the use of post-NAC MRI radiomics features can reduce the influence of confounding factors such as different treatment schemes and cycles in predicting apCR. In the future, we will consider adding pre-NAC radiomics features for further analysis.

## 5 Conclusion

As a preliminary attempt, this research included the MRI features of ALNs for radiomics analysis. The radiomics model based on post-NAC MRI features of breast tumors and ALNs showed good performance in predicting an apCR. We also combined the clinical factors and radiomics signature to build a clinical–radiomics model, which further improved the predictive ability for apCR. Finally, for future application in clinical practice, research is needed to test the feasibility of avoiding ALND and performing instead SLNB in patients predicted by the clinical–radiomics model to achieve an apCR.

## Data Availability Statement

The raw data supporting the conclusions of this article will be made available by the authors, without undue reservation.

## Ethics Statement

The studies involving human participants were reviewed and approved by the Ethics Committee of Peking University First Hospital. Written informed consent for participation was not required for this study in accordance with the national legislation and the institutional requirements.

## Author Contributions

LG, MM, XW, and JY: study design. LG, MM, JY, and XW: study conduct. LG, YL, QL, LXin, YC, LXu, XW, and JY: clinical data support. LG, MM, YJ, and XZ: data collection. LG, MM, XW, and XD: data processing. LG, MM, and XW: statistical data analysis. MM, NQ, YJ, XZ, and XW: MRI reading. LG and MM: drafting manuscript. All authors: revising and approving manuscript content. All authors contributed to editing the manuscript and read and approved the final version.

## Funding

This work was supported by the Beijing Medical Award Foundation (YXJL-2016-0040-0065), Beijing Medical Award Foundation (YXJL-2016-0040-0013), Youth Cultivation Fund of the Beijing Medical Award Foundation (20180502), and Interdisciplinary Clinical Research Project of Peking University First Hospital (2019CR38).

## Conflict of Interest

The authors declare that the research was conducted in the absence of any commercial or financial relationships that could be construed as a potential conflict of interest.

## Publisher’s Note

All claims expressed in this article are solely those of the authors and do not necessarily represent those of their affiliated organizations, or those of the publisher, the editors and the reviewers. Any product that may be evaluated in this article, or claim that may be made by its manufacturer, is not guaranteed or endorsed by the publisher.
