# Supplementary material for: A Clinical–Radiomics Model for Predicting Axillary Pathologic Complete Response in Breast Cancer With Axillary Lymph Node Metastases
Source: Front Oncol. 2021 Dec 21;11:786346. doi: 10.3389/fonc.2021.786346 (PMC8724774; doi:10.3389/fonc.2021.786346)
Supplement: Supplementary file 1 [file DataSheet_1.docx]

eTable 1. Radiomic features used in this study.

| Feature classification |  | Features names | |
| --- | --- | --- | --- |
| Shape Features (n=14) |  | Elongation |  |
|  |  | Flatness | |
|  |  | Least Axis Length | |
|  |  | Major Axis Length | |
|  |  | Maximum 2D Diameter (Column) | |
|  |  | Maximum 2D Diameter (Row) | |
|  |  | Maximum 2D Diameter (Slice) | |
|  |  | Maximum 3D Diameter | |
|  |  | Mesh Volume | |
|  |  | Minor Axis Length | |
|  |  | Sphericity | |
|  |  | Surface Area | |
|  |  | Surface Volume Ratio | |
|  |  | Voxel Volume | |
| First-order Statistical Features (n=18) |  | 10^th^ Percentile | |
|  |  | 90^th^ Percentile | |
|  |  | Energy | |
|  |  | Entropy | |
|  |  | Interquartile Range | |
|  |  | Kurtosis | |
|  |  | Maximum | |
|  |  | Mean Absolute Deviation | |
|  |  | Mean | |
|  |  | Median | |
|  |  | Minimum | |
|  |  | Range | |
|  |  | Robust Mean Absolute Deviation | |
|  |  | Root Mean Squared | |
|  |  | Skewness | |
|  |  | Total Energy | |
|  |  | Uniformity | |
|  |  | Variance | |
| Texture Features: GLCM (n=24) |  | Autocorrelation | |
|  |  | Cluster Prominence | |
|  |  | Cluster Shade | |
|  |  | Cluster Tendency | |
|  |  | Contrast | |
|  |  | Correlation | |
|  |  | Difference Average | |
|  |  | Difference Entropy | |
|  |  | Difference Variance | |
|  |  | Inverse Difference (ID) | |
|  |  | Inverse Difference Moment (IDM) | |
|  |  | Inverse Difference Moment Normalized (IDMN) | |
|  |  | Inverse Difference Normalized (IDN) | |
|  |  | Informational Measure of Correlation (IMC) 1 | |
|  |  | Informational Measure of Correlation (IMC) 2 | |
|  |  | Inverse Variance | |
|  |  | Joint Average | |
|  |  | Joint Energy | |
|  |  | Joint Entropy | |
|  |  | Maximal Correlation Coefficient (MCC) | |
|  |  | Maximum Probability | |
|  |  | Sum Average | |
|  |  | Sum Entropy | |
|  |  | Sum of Squares | |
| Textural Features: GLRLM (n=16) |  | Gray Level Non-Uniformity (GLN) | |
|  |  | Gray Level Non-Uniformity Normalized (GLNN) | |
|  |  | Gray Level Variance (GLV) | |
|  |  | High Gray Level Run Emphasis (HGLRE) | |
|  |  | Long Run Emphasis (LRE) | |
|  |  | Long Run High Gray Level Emphasis (LRHGLE) | |
|  |  | Long Run Low Gray Level Emphasis (LRLGLE) | |
|  |  | Low Gray Level Run Emphasis (LGLRE) | |
|  |  | Run Entropy (RE) | |
|  |  | Run Length Non-Uniformity (RLN) | |
|  |  | Run Length Non-Uniformity Normalized (RLNN) | |
|  |  | Run Percentage (RP) | |
|  |  | Run Variance (RV) | |
|  |  | Short Run Emphasis (SRE) | |
|  |  | Short Run High Gray Level Emphasis (SRHGLE) | |
|  |  | Short Run Low Gray Level Emphasis (SRLGLE) | |
| Textural Features: GLSZM (n=16) |  | Gray Level Non-Uniformity (GLN) | |
|  |  | Gray Level Non-Uniformity Normalized (GLNN) | |
|  |  | Gray Level Variance (GLV) | |
|  |  | High Gray Level Zone Emphasis (HGLZE) | |
|  |  | Large Area Emphasis (LAE) | |
|  |  | Large Area High Gray Level Emphasis (LAHGLE) | |
|  |  | Large Area Low Gray Level Emphasis (LALGLE) | |
|  |  | Low Gray Level Zone Emphasis (LGLZE) | |
|  |  | Size-Zone Non-Uniformity (SZN) | |
|  |  | Size-Zone Non-Uniformity Normalized (SZNN) | |
|  |  | Small Area Emphasis (SAE) | |
|  |  | Small Area High Gray Level Emphasis (SAHGLE) | |
|  |  | Small Area Low Gray Level Emphasis (SALGLE) | |
|  |  | Zone Entropy (ZE) | |
|  |  | Zone Percentage (ZP) | |
|  |  | Zone Variance (ZV) | |
| Textural Features: GLDM (n=14) |  | Dependence Entropy (DE) | |
|  |  | Dependence Non-Uniformity (DN) | |
|  |  | Dependence Non-Uniformity Normalized (DNN) | |
|  |  | Dependence Variance (DV) | |
|  |  | Gray Level Non-Uniformity (GLN) | |
|  |  | Gray Level Variance (GLV) | |
|  |  | High Gray Level Emphasis (HGLE) | |
|  |  | Large Dependence Emphasis (LDE) | |
|  |  | Large Dependence High Gray Level Emphasis (LDHGLE) | |
|  |  | Large Dependence Low Gray Level Emphasis (LDLGLE) | |
|  |  | Low Gray Level Emphasis (LGLE) | |
|  |  | Small Dependence Emphasis (SDE) | |
|  |  | Small Dependence High Gray Level Emphasis (SDHGLE) | |
|  |  | Small Dependence Low Gray Level Emphasis (SDLGLE) | |

(GLCM: Gray Level Co-occurrence Matrix; GLRLM: Gray Level Run-length Matrix; GLSZM: Gray Level Size Zone Matrix; GLDM: Gray Level Dependence Matrix.)

**S1:**  **Details of the radiomics modeling pipelines**：

Normalization：Mean-Normalizer

The function of Mean-Normalization is to reduce the fluctuation of the sample data so that the gradient descent can find a ‘shortcut’ more quickly to reach the global minimum. Make the sample data has also changed significantly in a smaller range. The function is$: x^{*}=\frac{x-\mu}{max-min}$ (μ is the mean of all sample data).

Normalization: Z-score

This method gives the mean and standard deviation of the original data to standardize the data. The processed data conforms to the standard normal distribution, that is, the mean is 0, the standard deviation is 1, and the conversion function is: $x^{*}=\frac{x-\mu}{\delta}$ (μ is the mean of all sample data, and σ is the standard deviation of all sample data).

Normalization: Min-Max

Min-Max normalization is also known as dispersion standardization, it is a linear transformation of the original data to map the result value to [0-1]. The max is the maximum value of the sample data, and the min is the minimum value of the sample data. The conversion function is$: x^{*}=\frac{x-min}{max-min}$

Dimension reduction: PCC

PCC is used to measure the degree of linear correlation between two features, if the PCC value of the two features was larger than 0.9, one of the features will be removed randomly. In this way, the dimension of the features is reduced and the features still retain the original classification.

Dimension reduction: PCA

PCA is a mathematical dimension reduction method that uses an orthogonal transformation to convert a series of linearly related variables into a set of new linearly unrelated variables. The meaning of these data is different from the original data, but contains most of the previous data, and has a lower dimension, which is convenient for further analysis.

Feature selection: RFE

Recursive feature elimination (RFE) is a common feature selection method. It works by removing features recursively and building a model on the remaining features. It uses the accuracy of the model to determine which features (or combinations of features) contribute more to the prediction results.

Feature selection: ANOVA

Analysis of variance (ANOVA) is used to explore the significant features corresponding to the labels. The F-value was calculated to evaluate the relationship between the features and the label. The features were sorted by the corresponding F-value and a specific number of features were selected to build models.

Feature selection: KWT

The Kruskal-Wallis test is used to explore the significant features of the ROIs, and all features have corresponding P-values. If the P-values are smaller than 0.05, the features are thought significant to the corresponding label and will be further analyzed.

Feature selection: Relief

Relief is a feature weighting algorithm, which assigns different weights to features according to the correlation of each feature to a category, then features whose weight is less than a certain threshold will be removed.

Classifier: LASSO

Least Absolute Shrinkage and Selection Operator (LASSO) is a linear regression method that uses L1 regularization. Using L1 regularization will make the weight of some learned features be 0, so as to achieve the purpose of sparsity and feature selection.

Classifier: RF

Random Forest (RF) is composed of many decision trees, and there is no correlation between different decision trees. When we perform classification tasks, new samples are inputted, and each decision tree in the forest begins to judge and classify separately. Each decision tree will get its own classification result. The random forest will treat the most classification result in the decision trees as the final result.

Classifier: SVM

Support Vector Machine (SVM) is a generalized linear classifier that binary classification of data according to supervised learning. Its decision boundary is the maximum margin for solving the learning sample. Hyperplane. SVM uses the hinge loss function to calculate empirical risk and adds a regularization term to the solution system to optimize structural risk. It is a classifier with sparsity and robustness. SVM can perform non-linear classification through the kernel method, which is one of the common kernel learning methods.

Classifier: Decision Tree

Decision tree is a tree built on the basis of strategic choices. In machine learning, a decision tree is a predictive model; it represents a mapping relationship between object attributes and object values. Each node in the tree represents an object, and each bifurcation path represents a possible attribute value. The path from the root node to the leaf node corresponds to a judgment test sequence. The decision tree can be a binary tree or a non-binary tree, it can also be regarded as a set of if-else rules, or it can be regarded as a conditional probability distribution in a feature space. The knowledge acquired by the decision tree through training directly forms a hierarchical structure. This structure preserves and displays knowledge in such a way that even non-experts can easily understand it.

Classifier: XGBoost

XGBoost is one of the boosting algorithms. The idea of Boosting algorithm is to integrate many weak classifiers to form a strong classifier. Because XGBoost is a boosted tree model, it integrates many tree models to form a strong classifier. The tree model used is the CART regression tree model.

Classifier: Adaboost

Adaboost is an iterative algorithm. Its core idea is to train different classifiers (weak classifiers) for the same training set, and then combine these weak classifiers to form a stronger final classifier (strong classifier).

Classifier: LR

Logistic Regression (LR) is mainly used for binary classification problems, using the Logistic function (or Sigmoid function). The value range of the independent variable is (-INF, INF) and the value range of the independent variable is (0,1). The function form is$: g(z)=\frac{1}{1+e^{-z}}$

eTable 2. Multivariable logistic regression analysis of predictors for apCR in the training set.

| Characteristic | OR | 95% CI | P value |
| --- | --- | --- | --- |
| Radiomics signatures | 493.5 | 6.55-6173.4 | <0.001 |
| Clinical N stage | 4.14 | 1.28-14.85 | 0.022 |
| HR  Clinical tumor response | 3.82  2.90 | 1.29-12.48  4.43-22.7 | 0.019  0.28 |

(OR: odds ratio; CI: confidence interval; HR: Hormone receptor; HER2: Human epidermal growth factor receptor 2; SD: stable disease; PR: stable disease; CR: complete response; T: tumor; N: node.)
